# Explainable Artificial Intelligence Models for Predicting Depression Based on Polysomnographic Phenotypes

**DOI:** 10.3390/bioengineering12020186

**Published:** 2025-02-15

**Authors:** Doljinsuren Enkhbayar, Jaehoon Ko, Somin Oh, Rumana Ferdushi, Jaesoo Kim, Jaehong Key, Erdenebayar Urtnasan

**Affiliations:** 1 Department of Biomedical Engineering, Yonsei University, Wonju 26493, Republic of Korea; doji0704@yonsei.ac.kr (D.E.); jaehoonko@yonsei.ac.kr (J.K.); bliss220@yonsei.ac.kr (S.O.); rumanayonsei@yonsei.ac.kr (R.F.); 2 Division of Semiconductor System Engineering, Yonsei University, Wonju 26493, Republic of Korea; jaekim2030@yonsei.ac.kr; 3 Yonsei Institute of AI Data Convergence Science, Yonsei University, Wonju 26493, Republic of Korea; 4 Department of Medical Engineering, Huree University of ICT, Ulaanbaatar 16061, Mongolia

**Keywords:** explainable artificial intelligence, machine learning, depression, polysomnographic phenotypes

## Abstract

Depression is a common mental health disorder and a leading contributor to mortality and morbidity. Despite several advancements, the current screening methods have limitations in enabling the robust and automated detection of depression, thereby hindering early diagnosis and timely intervention. This study aimed to develop explainable artificial intelligence (AI) models to predict depression using polysomnographic phenotype data, ensuring high predictive performance while providing clear insights into the importance of features influencing the risk of depression. Advanced machine learning algorithms such as random forest, extreme gradient boosting, categorical boosting, and light gradient boosting machines were employed to train and validate the predictive AI models. Phenotype data from subjective health questionnaires, clinical assessments, and demographic factors were analyzed. The explainable AI models identified the important features, and their performance was evaluated using cross-validation. The study population, comprising 114 control participants and 39 individuals with depression, was stratified based on validated depression-scoring methods. The proposed explainable AI models achieved an F1-score of 85%, verifying their high reliability in predicting depression. Key features influencing the risk of depression, such as anxiety disorders, sleep efficiency, and demographic factors, offer actionable insights for clinical practice, highlighting the transparency of these models. This study proposed and developed explainable AI models based on polysomnographic phenotype data for the automated detection of depression and verified that these models help improve mental health diagnostics, enabling timely interventions.

## 1. Introduction

Depression is a common mental health condition that significantly affects the quality of life and leads to neurological impairments and cognitive sluggishness. According to the data from the World Health Organization, approximately 280 million people worldwide are affected by this debilitating condition, suggesting a global health crisis [1]. Tragically, approximately 720,000 individuals die each year of depression [2] with up to 53% of the suicides occurring among those affected by this condition [3]. Thus, depression has become the leading cause of death among various mental disorders owing to its high mortality and morbidity rates. Additionally, compared to healthy individuals, patients with depression are at an increased risk of developing chronic conditions, including cancer, neurological disorders, and cardiovascular diseases [4]. These statistics emphasize the severe effects of depression and highlight the urgent need for effective diagnostic and interventional strategies.

Sleep disturbance is a prominent symptom and contributing factor among the numerous factors contributing to the burden of depression. A well-established correlation exists between poor sleep quality and mood disorders, with depression symptoms often manifesting after nights of disrupted sleep [5]. Meta-analyses have confirmed that sleep disturbances, specifically insomnia, are significant contributors to the emergence and progression of major depressive disorders [6]. Furthermore, therapeutic approaches targeting sleep disorders have shown notable benefits in alleviating depressive symptoms [7]. However, the intricate two-way connection between depression and sleep disturbances challenges conventional diagnostic approaches. Some individuals with sleep disorders may not exhibit depressive symptoms, whereas others with normal sleep patterns may exhibit depressive phenotypes. This variability highlights the need for explainable AI models to accurately predict depression, emphasizing the potential of phenotypic data to provide deeper insights [8].

Conventional diagnostic methods for depression, including self-reported health questionnaire (SHQ) [9] and patient health questionnaire (PHQ-9) [10], are largely based on subjective self-reports and clinician interpretations. Although widely used in clinical practice, these methods are limited by their inherent subjectivity, variability in clinical expertise, and the possibility that patients may conceal or under-report symptoms. Therefore, diagnosing depression remains challenging. Early diagnosis and timely intervention are critical, particularly because of the high conversion rate from depressive disorders to major depressive disorders and their substantial effect on the economic and mental well-being of individuals [11]. These challenges emphasize the need for more objective and efficient diagnostic approaches, and machine learning integrated with polysomnographic (PSG) phenotype data can play an important role in this regard.

With the rapid advancements in artificial intelligence (AI), its applications have expanded across numerous fields, significantly influencing modern society. Machine learning, a core pillar of AI, excels in recognizing and classifying complex patterns in massive datasets. This ability makes it particularly suitable for analyzing the vast and intricate data generated in healthcare [12]. Consequently, machine learning techniques are increasingly being applied in the healthcare and medical fields. Owing to the complexity of the variables influencing depression and their interrelationships, prediction models developed using machine learning techniques are well positioned to reliably predict depressive disorders [13].

Recent studies have shown that employing feature selection methods can significantly enhance the performance of depression-prediction models. For instance, Hassan et al. [14] emphasized the usefulness of EEG-based features for depression detection using feature selection methods combined with models such as random forest (RF), extreme gradient boosting (XGBoost), categorical boosting (CatBoost). This highlights the importance of feature selection in improving the classifier performance by identifying the most relevant EEG features for predicting depression. Li et al. [15] applied tree-based ensemble algorithms, including XGBoost, LightGBM, and CatBoost, to predict the risk of depression, and used Shapley’s additive explanations (SHAP) for model interpretability. This approach provides insights into the key factors influencing depression, making it highly relevant for research on depression prediction. Similarly, Fan et al. [16] focused on EEG data and compared the performances of machine learning models in selecting relevant features for depression detection. These studies demonstrate the importance of identifying meaningful predictors to improve the model’s performance.

Sharma et al. [17] extended the application of machine learning to depression classification by utilizing biomarkers from the Lifelines database. Their use of XGBoost effectively addressed dataset imbalances and yielded a highly balanced accuracy for depression prediction. Thus, both their study and our study reinforce the utility of ensemble models such as XGBoost for precise classification in depression prediction, especially when working with complex and imbalanced datasets. Wang et al. [18] utilized a stacking approach combining LightGBM, CatBoost, and RF to handle big data modeling and demonstrated how these models can be used effectively for depression prediction. This highlights the effectiveness of combining multiple models to improve prediction accuracy. Singh et al. [19] compared different machine learning models including RF, XGBoost, LightGBM, and CatBoost to predict depression, anxiety, and stress. Their comprehensive comparison of the model performances provides valuable insights into identifying the algorithms that offer the best results for mental health prediction.

Herein, we propose an explainable AI model designed to detect depression using the PSG phenotypic data from the Best Apnea Interventions in Research (BESTAIR) study. By leveraging advanced AI techniques, including RF, XGBoost, CatBoost, and LightGBM, we integrated phenotype data to create a robust clinical decision support system for predicting depression. Our models use longitudinal sleep research data from the BESTAIR study, provided by the National Sleep Research Resources (https://sleepdata.org/datasets/bestair, accessed on 3 May 2023) [20], to enhance diagnostic accuracy and facilitate early intervention in clinical practice.

Unlike previous work, our study prioritizes explainability and transparency, which are critical for clinical adoption. Traditional AI models are often regarded as “black boxes”, limiting their interpretability. To address this, we integrate Shapley’s additive explanations (SHAP) to provide insights into key predictive features, enhancing trust and usability for clinicians. This study addresses crucial contributions in the existing literature by the following:

1. Shifting from subjective report-based models to objective PSG phenotype data, improving diagnostic accuracy.
2. Developing explainable AI models that offer transparency and actionable insights in contrast to the opaque models previously used.
3. Integrating sleep research with machine learning to better understand the interplay between sleep characteristics and depression, leading to more comprehensive clinical interventions.

To provide a clear understanding of our approach, the subsequent section of this paper is organized as follows: Section 2 discusses the methodology, including the machine learning algorithms used, the data processing techniques, and the validation strategies employed to develop the predictive models. Section 3 presents the results obtained from the model’s performance metrics, the key features identified, and the insights gained from the analysis. Section 4 provides a discussion of the results, comparing them with the existing literature and exploring the potential clinical applications of the developed explainable AI models. Ultimately, the study concludes in Section 5 by summarizing the outcomes and suggesting directions for future research to enhance AI’s application in depression prediction and mental health diagnostics.

## 2. Materials and Methods

### 2.1. Study Population

**Figure 1:**
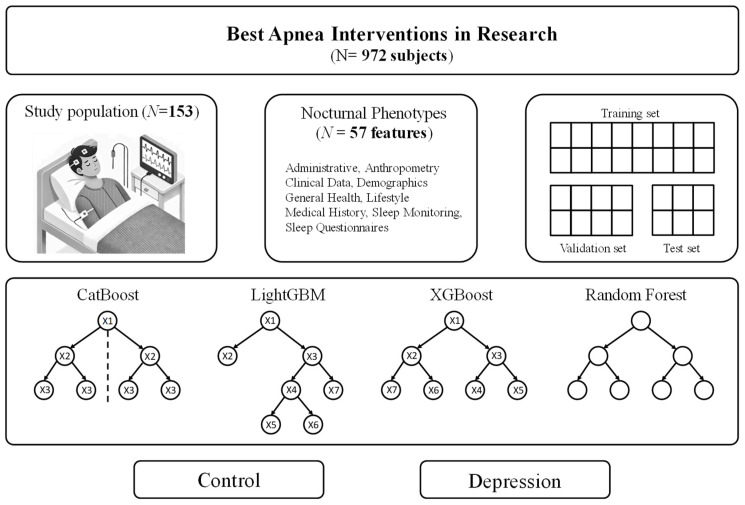
Overview of the proposed depression-prediction framework.

This study used data from the BESTAIR trial, a cohort study investigating the relationship between sleep disturbances and depression (Figure 1). The cohort comprised 153 participants, including 39 patients diagnosed with depression and 114 matched controls. To ensure balanced comparisons, the data were stratified according to the (self-reported physician diagnosis) SHQ depression scores. The participants were tracked from 2011 to 2014, and the phenotype data were collected during baseline visits. The dataset included PSG, SHQ, and treatment data. PSG provided objective sleep measurements, while the SHQ and other questionnaires offered insights into the participants’ mental health.

Additionally, physiological data, such as sleep efficiency, apnea–hypopnea index (AHI), systolic blood pressure, and anthropometric measurements (e.g., neck and waist circumferences), were gathered. Biochemical data, including the total cholesterol, insulin, albumin, creatinine, and triglycerides, were also recorded. PSG was used to assess the AHI, which represents the hourly occurrence of apnea and hypopnea events. This comprehensive dataset supported the development of an explainable AI model for predicting depression (Table 1).

### 2.2. PSG Phenotypes

The dataset comprised 153 observations, including 39 cases of depression and 114 controls (Table 1), with a total of 57 parameters grouped into three primary categories: (1) polysomnography, with 12 parameters providing detailed, objective measurements of sleep disturbances; including sleep efficiency, (Rapid Eye Movement) REM latency, apnea-hypopnea index (AHI), and oxygen desaturation index (ODI), which are critical for assessing sleep quality and potential disturbances linked to depression; (2) treatment, containing six parameters related to therapeutic approaches for depression such as CPAP (Continuous Positive Airway Pressure) adherence, use of nasal dilators, and other intervention-related metrics—they reflect how treatments aimed at improving sleep can impact depressive symptoms; and (3) questionnaires, which include 39 parameters based on SHQ data, and these parameters assess various mental health conditions, including anxiety disorder, overall mental health scores, and subjective sleep quality. These self-reported metrics provide insight into the psychological and behavioral aspects of sleep and depression (Table 2). These diverse phenotypes offer a comprehensive view of both sleep and treatment characteristics, which are essential for investigating the complex relationship between sleep disturbances and depression.

### 2.3. Explainable AI Models

This study used machine learning techniques to evaluate the significance of various parameters and identify the most influential factors associated with depression. Four advanced algorithms—RF, XGBoost, CatBoost, and LightGBM—were applied to extract the most relevant and impactful features from the PSG phenotype data. Each algorithm highlights distinct parameters that provide valuable insights into the dataset.

#### 2.3.1. Random Forest

RF is a widely used and advanced ensemble algorithm in machine learning that typically produces excellent outcomes without extensive hyperparameter tuning. Although overfitting can be a significant issue, the classifier is less likely to overfit if there are sufficient trees in the forest. The RF algorithm can also handle missing data issues. Generating diverse trees using techniques such as bootstrap aggregation (bagging) and feature randomness reduces overfitting and improves generalization. Each tree is trained on a random subset of data and features, enabling the algorithm to manage complex relationships in the datasets while providing reliable predictions in both the classification and regression tasks. This is because of its versatility, resistance to overfitting, and ability to handle large datasets [21]. The Gini importance for feature j in RF is calculated as [22]

I_G_ (j) = ∑ T ∈ forest ∑ _t ∈ T_ ∆i (S_t_, j), (1)

where Δi (S_t_, j) is the decrease in impurity for feature j at node t.

#### 2.3.2. XGBoost

XGBoost is an open-source machine learning framework that uses supervised learning to predict the outcomes, relying on multiple decision trees within the library to make predictions. The algorithm is trained using batch learning, followed by a model-based approach to generalize the results. It constructs models using all the available data to establish relationships between predictions and outcomes, which are then applied to the test data. The term “extreme” highlights the algorithm’s focus on maximizing the computational efficiency. XGBoost uses gradient boosting to enhance the performance of weak models, making it highly effective for both the regression and classification tasks in various machine learning applications [23]. XGBoost uses gradient boosting and provides feature importance based on the gain. The gain for a feature is calculated as [22]

Gain = 1/2 [G_L_^2^/H_L_ + G_R_^2^/H_L_ − (G_L_ + G_R_)^2^/(H_L_ + H_R_)] − λ *×* (H_L_ + G_R_) − γ, (2)

where G and H are the sum of the first- and second-order gradients, respectively, and λ and γ are regularization terms.

#### 2.3.3. LightGBM

LightGBM is an ensemble-learning algorithm designed to build decision trees, which provide faster and more efficient performance than XGBoost. It consumes less memory, delivers higher accuracy, and supports parallel computing. The key features of LightGBM include gradient-based one-sided sampling (GOSS), exclusive feature bundling (EFB), and a histogram-based algorithm for differential acceleration. The synergy of these techniques enhances the performance and computational efficiency of the algorithm [24]. The GOSS technique retains samples with large gradients while randomly selecting a proportion of samples with smaller gradients based on their size. The EFB algorithm combines two non-mutually exclusive features. This approach reduces both the number of features and the time complexity, thereby enhancing the computational efficiency of the model.

#### 2.3.4. CatBoost

CatBoost is a powerful open-source machine learning algorithm from the gradient-boosted decision tree (GBDT) family. It addresses the limitations of the traditional gradient-boosting models such as target leakage and prediction shifts. Notably, CatBoost excels in handling categorical features through ordered boosting, distinguishing it from other GBDTs such as XGBoost and LightGBM. Unlike deep learning models, CatBoost performs well with limited training data and computational resources [25].

### 2.4. Experimental Setup

This study employed Python (version 3.11.4; Python Software Foundation, Fredericksburg, VA, USA) as the primary open-source programming language for all the tasks. Statistical analysis was conducted using SciPy [26] (version 1.8.1), and the scikit-learn [27] library (version 1.1.2) was used to develop the predictive models. The training and testing of each model were performed on hardware equipped with GTX 1080 Ti (3584 CUDA core) running on Windows 10. To enhance the efficiency of the training and testing processes, the dataset was partitioned into minibatches, each consisting of 256 segments. An accumulated gradient was computed for the parameters after each minibatch was trained.

### 2.5. Evaluation Metrics

In this study, a depression-prediction model was trained and evaluated using four-fold cross-validation. The performance of various algorithms on the testing dataset was assessed after completing the training and validation phases. The model performance was then compared using a confusion matrix for a binary classification problem [28]. Based on the information provided by the confusion matrix, the following performance metrics were calculated: accuracy, precision, recall (sensitivity), specificity, F-measure, and receiver operating characteristic (ROC) area [29,30]. These metrics were computed using Equations (1)–(4).

Accuracy = (TP + TN)/(TP + TN + FP + FN)(3)

Sensitivity is the probability of correctly identifying individuals who have the disease.

Sensitivity (Recall) = TP/(TP + FN)(4)

Specificity is the probability of correctly identifying individuals who do not have the disease, without false positives.

Specificity = TN/(TP + FP)(5)

Precision represents the positive predictive value, which indicates the proportion of positive results that are true positives.

Precision = TP/(TP + FP)(6)

ROC curves are two-dimensional graphs in which the true positive rate is plotted on the *Y*-axis and the false positive rate is plotted on the *X*-axis. A ROC curve illustrates the tradeoffs between the benefits (true positives) and costs (false positives) of a diagnostic test [31].

## 3. Results

The performance of the proposed depression model was evaluated through experiments, and meaningful results were obtained based on the baseline characteristics of the study population (Table 1). The study population comprised 65.4% males and 35.6% females, with a nearly 2:1 male–female ratio in the depression group. A significant difference in sex distribution was observed between the depression and control groups (*p* < 0.001). The age distribution showed a higher proportion of participants aged 46–59 years (7.2%) and 59–66 years (9.8%) in the depression group than in the control group, with a significant age difference between the groups (*p* < 0.001), suggesting that middle-aged individuals may be at higher risk for depression due to a combination of physiological, psychological, and social factors.

As hypothesized, the depression group exhibited significantly lower sleep efficiency (76.6 ± 13.8 h) than the control group (87.0 ± 9.8 h, *p* < 0.001), highlighting the strong association between sleep disturbances and depression. Furthermore, the depression group had a higher AHI (33.5 ± 22.6 per h) than the control group (28.7 ± 14.6 per h), and this difference did not reach statistical significance (*p* > 0.01). However, this trend suggests a potential association that warrants further investigation. Other measures, including body mass index (BMI), blood pressure, waist circumference, neck circumference, and biochemical markers (e.g., total cholesterol, triglycerides, creatinine, fasting insulin, and albumin–creatinine ratio), did not show significant differences between the groups (*p* > 0.01).

### 3.1. Correlation Between PSG Phenotypes and Depression

**Figure 2:**
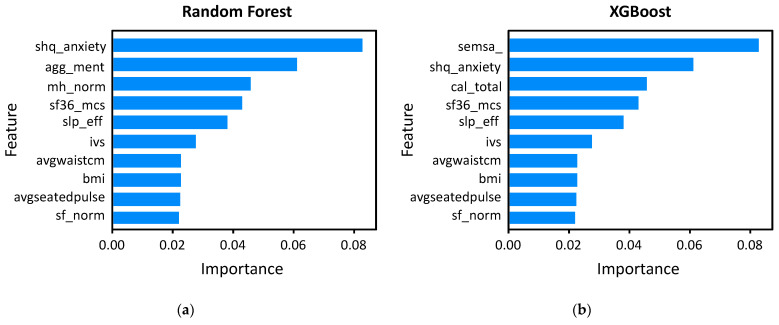

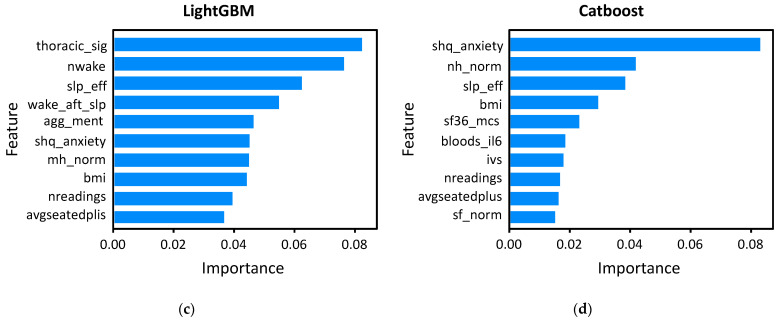
Feature importance plots for four machine learning models used to predict early depression based on phenotypic data: (**a**) RF, (**b**) XGBoost, (**c**) LightGBM, and (**d**) CatBoost. Each plot displays the important features contributing to the model’s prediction, ranked by their importance scores. These models highlight the key factors (e.g., anxiety disorder, sleep efficiency, and mental health score) and physiological markers (e.g., BMI and pulse rate) that are associated with depression prediction.

The feature importance analysis used four machine learning models: RF, XGBoost, LightGBM, and CatBoost (Figure 2). Across all the models, several features consistently emerged as significant predictors of early depression. Notably, mental health-related variables, such as the self-reported physician diagnosis of anxiety disorder (shq_ anxiety disorder), aggregated mental health scores (agg_ment), and sleep efficiency (slp_eff), were among the top contributors. The RF model highlighted the shq_anxiety disorder as the most influential feature, while the XGBoost and LightGBM models emphasized the importance of sleep-related variables such as insomnia (shq_insomnia) and sleep apnea. The CatBoost model reinforced the significance of physiological markers such as BMI and pulse rate. These results indicate that both the mental health metrics and physical health factors play critical roles in accurately predicting early depression across the different machine learning models.

Using explainable AI, we further optimized the model’s performance by comparing its accuracy with all the features against a model utilizing the top 10 selected features through feature importance analysis. The optimized model maintained similar predictive accuracy while improving computational efficiency. As shown in Table 3, the feature analysis highlights these top 10 features, demonstrating their contribution to the model’s predictive power and reliability, along with their descriptions, which emphasize their relevance to both the mental and physical health metrics.

### 3.2. Performance Evaluation of the Proposed Depression-Prediction Model

The performances of the four machine learning models (RF, XGBoost, LightGBM, and CatBoost) in predicting early depression are summarized in Table 4. RF and CatBoost demonstrated the highest overall performances, achieving an accuracy of 85.0% along with matching precision, recall, and F1-score of 85.0%. XGBoost achieved an accuracy of 83.0%, with precision, recall, and F1-score of 83.0%, while LightGBM had an accuracy of 83.0%, with a slightly lower recall (83.0%) but similar precision and F1-score of 86.0% and 85.0%, respectively. In terms of the area under the curve (AUC), RF outperformed the others, with an AUC of 83.0%, followed by CatBoost (79.0%), LightGBM (77.0%), and XGBoost (71.0%). Overall, RF and CatBoost were the most reliable models for early depression prediction, while XGBoost showed relatively weaker performance.

For comparison, the Artificial Neural Network (ANN) was included as a baseline model, demonstrating lower performance across all the metrics. In contrast, our explainable AI models outperformed the ANN, achieving significantly higher precision, recall, F1-score, and accuracy, highlighting their superior capability in predicting early depression.

### 3.3. Confusion Matrix Analysis

**Figure 3:**
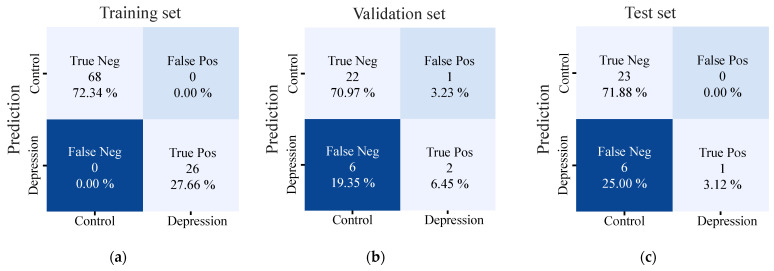
Confusion matrices for (**a**) training, (**b**) validation, and (**c**) test sets. The matrices illustrate the performance of the prediction model in distinguishing between the control and depression cases. The percentage value represents the proportion of correct or incorrect predictions for each category.

The confusion matrices shown in Figure 3 summarize the classification performance of the machine learning models in predicting depression across the training, validation, and test sets. For the validation set, the model exhibited slight misclassifications, with one control instance misclassified as depression and two cases misclassified as controls. This resulted in an accuracy of 70.97% and 19.35% for the control and depression groups, respectively. Similarly, for the test set, the model correctly classified 23 control cases (71.88%) and 1 depression case (3.12%), while 8 depression instances (25.00%) were misclassified as controls. We monitored the model’s performance across the training, validation, and test sets and observed no signs of overfitting or underfitting. The model’s performance was consistent, with only slight accuracy variations, indicating good generalization. While the accuracy dropped slightly in the validation and test sets, especially for the depression cases, this highlights room for improvement in handling minority classes. These results reflect the high model performance in distinguishing between the control and depression cases, although the accuracy decreased slightly in the validation and test sets, particularly for the depression cases, suggesting the scope for improvement in handling minority classes.

**Figure 4:**
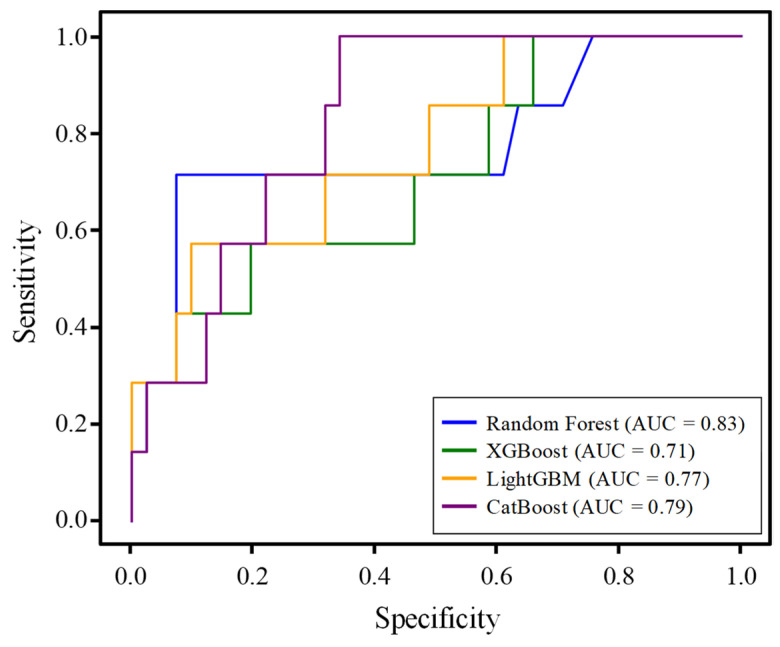
Comparison of the area under the ROC curves (AUC) for the depression-prediction models. The ROC curves illustrate the performance of the four machine learning models—RF, XGBoost, LightGBM, and CatBoost—in predicting depression. The AUC values reflect the ability of the model to distinguish between positive and negative cases, with higher AUC values indicating better discriminatory power. The RF model demonstrated the highest AUC, followed by CatBoost, LightGBM, and XGBoost.

The ROC curves of the four depression-prediction models—RF, XGBoost, LightGBM, and CatBoost—showed varying levels of performance in discriminating between the depressed and non-depressed individuals (Figure 4). Among the models, RF exhibited the highest AUC, indicating a superior ability to correctly classify depression cases. CatBoost followed closely, demonstrating a strong performance with a high AUC value. XGBoost and LightGBM also showed reasonable performances, although their AUC values were slightly lower, suggesting weaker discrimination capabilities than those of RF and CatBoost. The ROC curves highlight the tradeoffs between sensitivity and specificity for each model, providing valuable insights into their effectiveness in depression prediction. These findings suggest that RF and CatBoost may be the most promising models for achieving high predictive accuracy in clinical settings. In contrast, other models, although effective, may require further optimization to achieve similar performance levels.

### 3.4. Parameter Optimization

In this study, we evaluated the performance of explainable AI models, RF, XGBoost, LightGBM, and CatBoost by optimizing their key parameters to achieve optimal classification accuracy (Table 5). To prevent overfitting, we specifically focused on fine-tuning crucial parameters.

## 4. Discussion

This study developed explainable AI models for predicting depression using the PSG phenotype data by employing advanced machine learning techniques, namely, RF, LightGBM, XGBoost, and CatBoost. These models were carefully designed and optimized to establish a stable and accurate prediction framework that could primarily address the challenges associated with interpreting depression data. A multi-site sleep study dataset from the BESTAIR database was used to train, validate, and evaluate the proposed approach. We achieved notable results in distinguishing the depression cases from the healthy cases using self-reported data, with a mean accuracy of 85.0%. These findings are consistent with those of previous studies that utilized similar machine learning models for depression prediction, as summarized in Table 6.

While several traditional studies have proposed methods for predicting depression using different datasets, all of these listed studies relied on EEG signals or biomarker-based data, whereas our study used PSG phenotype data. Therefore, it may not be entirely appropriate to directly compare the performances of these studies with our results. However, we reviewed and analyzed these recent studies to gain insights and identify the current state-of-the-art approaches in depression prediction. These studies have highlighted the growing effectiveness of machine learning in mental health assessments, further validating the results and methodology of our model.

For example, Sharma et al. [17] achieved an accuracy of 97.6% using XGBoost, while Singh et al. [19] and Hassan et al. [14] achieved accuracies of 94.0% with SVM and 93.5% with SVM and SVM-RFE, respectively. In contrast, our model employing RF, LightGBM, XGBoost, and CatBoost achieved accuracy, precision, recall, and F1-score of 85.0%, which is slightly lower than those of some studies. While slightly lower than some studies, our results align with the trend of ensemble models balancing precision and recall in the complex domain of mental health prediction.

While our study focused on ensemble methods, such as RF, XGBoost, LightGBM, and CatBoost, Artificial Neural Networks (ANNs) have also been explored for depression prediction. ANNs, including Recurrent Neural Networks (RNNs) and Convolutional Neural Networks (CNNs), can capture complex patterns in large datasets and have shown promise in mood disorder prediction and affective state recognition. However, they require significant computational resources, are prone to overfitting with limited data, and lack interpretability, which is crucial for clinical applications. In contrast, the ensemble methods in this study strike a better balance between performance and interpretability, providing feature importance insights that help clinicians understand the predictive factors. Future research could combine ANNs with ensemble models, leveraging their strengths to enhance prediction accuracy while maintaining interpretability for clinical use.

**Figure 5:**
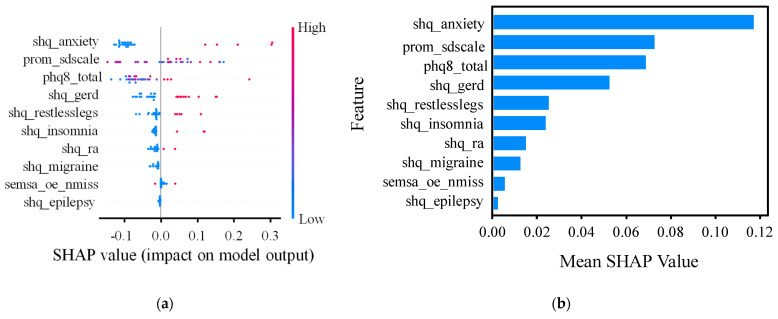
Shapley additive explanations (SHAP): (**a**) The standard bar chart shows the contribution of each feature to the prediction of depression using the RF model. The features are ranked by the sum of the SHAP values across all instances, highlighting their contributions to the model’s outputs. (**b**) The classified bar chart illustrates the distribution of the effects of each feature on the RF model outputs. Each point represents a SHAP value for a feature in a single instance, with the color gradient indicating the feature value (red for higher values and blue for lower values). Features are ranked in order of importance, with the top features contributing the most to the model’s predictions. RF, random forest; shq_anxiety, anxiety disorders; prom_sdscale, sleep disturbance; phq8_total, total score of the patient health questionnaire (PHQ); shq_gerd, gastroesophageal reflux disease; shq_restlesslegs, Periodic Leg Movements; shq_ra, Rheumatoid Arthritis; semsa_oe_nmiss, number of missing items of outcome expectancies subscale; and shq_epilepsy, self-reported epilepsy diagnosis.

Notably, while RF, XGBoost, LightGBM, and CatBoost models indicated that anxiety disorders and sleep disturbances were the most significant predictors for depression, the SHAP analysis revealed a different feature importance (Figure 5). Specifically, PHQ scores demonstrated the strongest predictor for depression, as indicated by the SHAP summary plot. The SHAP values were averaged across all the patients, providing a more comprehensive assessment of feature importance. This distinction is critical, as features with a lower individual impact but higher prevalence, such as mild sleep disturbances, may exhibit low odds ratios in traditional statistical models but high SHAP values. Consequently, the SHAP analysis enabled a more nuanced understanding of how features influence depression prediction across a broader population, offering valuable insights into the underlying mechanisms that conventional methods may overlook.

Feature selection is a key factor in improving performance and interpretability. Other studies have used advanced techniques such as maximum information coefficient, wrapper methods, and importance ranking to identify key predictors such as self-perceived mental health and socioeconomic factors. In our study, we prioritized interpretability by identifying key features, such as anxiety disorders, sleep efficiency, and demographic factors, which contributed a significant role in predicting depression, providing clinicians with actionable insights, and enhancing trust in AI-driven mental health assessments.

Despite these promising results, challenges remain in ensuring data quality, model generalizability, and clinical integration. Future research should focus on developing more robust and generalizable models by integrating diverse multimodal datasets. Longitudinal studies are also necessary to assess the long-term effect of machine learning on depression prediction and intervention.

One advantage of our study is the use of four different machine learning models, allowing for a broader performance evaluation. The proposed AI model enhances real-world relevance by including personal experiences that are crucial for mental health diagnoses using PSG phenotypic data. Explainable AI ensures transparency, helps clinicians interpret predictive factors, and increases clinical adoption. Finally, the scalability of ensemble methods such as RF and XGBoost makes our approach scientifically innovative and feasible for real-world mental health assessments.

## 5. Conclusions

This study developed explainable AI models for depression detection using PSG phenotype data and a combination of machine learning algorithms including RF, XGBoost, LightGBM, and CatBoost. The proposed model effectively distinguished patients with depression from healthy individuals with a mean accuracy of 85.0% based on the data from the BESTAIR sleep study. Statistical analysis revealed that the model’s performance metrics (accuracy, precision, recall, and F1-score) were statistically significant, with *p*-values < 0.05 for all the comparisons against the baseline models, indicating that the proposed approach outperforms the traditional methods. This demonstrates the potential of the proposed model as a robust tool for depression prediction. Our findings support the use of AI-driven techniques for mental health assessment and offer valuable insights into the mental well-being of individuals.

In the future, we will aim to enhance the model by integrating multimodal data, such as behavioral and physiological signals, to improve the precision and generalizability of depression prediction across diverse populations.

## Figures and Tables

**Table 1:** Demographic and anthropometric characteristics of the study cohort.

Measures	Total	Control	Depression	*p*-Value
Participants (*N*)	153 (100)	114 (74.5)	39 (25.5)	
Sex				
Female	53 (34.6)	33 (21.6)	20 (13.1)	
Male	100 (65.4)	81 (52.9)	19 (12.4)	<0.001
Height (cm)	171.86 ± 9.43	170.5 ± 6.2	168.75 ± 5.8	NS
BMI (kg/m^2^)	31.1 ± 5.3	31.11 ± 5.2	32.25 ± 6.5	NS
AHI (per hour)	29.9 ± 17.1	28.7 ± 14.6	33.5 ± 22.6	NS
Age (years)				
46–59	40 (26.1)	29 (18.9)	11 (7.2)	<0.001
59–66	37 (24.2)	22 (14.4)	15 (9.8)	
66–69	40 (26.1)	33 (21.6)	7 (4.6)	
70–76	36 (23.5)	30 (19.6)	6 (3.9)	
Blood Pressure (mmHg)				
Systolic	126.2 ± 17.2	125.3 ± 16.9	128.7 ± 17.9	NS
Diastolic	70.6 ± 9.3	70.4 ± 9.0	71.0 ± 10.2	
Sleep efficiency (hour)	84.4 ± 11.8	87.0 ± 9.8	76.6 ± 13.8	<0.001
Waist circumference (cm)				
Female	112.5 ± 17.1	112.3 ± 15.6	114.6 ± 19.8	NS
Male	109.4 ± 12.1	108.4 ± 11.7	112.8 ± 12.9	
Neck circumference (cm)				
Female	38.5 ± 3.6	38.2 ± 3.4	39.4± 4.1	NS
Male	43.1 ± 3.1	43.05 ± 3.0	43.7 ± 3.3	NS
Total cholesterol (mg/dL)	165.4 ± 36	163.1 ± 33.2	171.8 ± 43.1	NS
Triglycerides (mg/dL)	128.9 ± 75.6	126.2 ± 68.4	136.9 ± 93.8	NS
Creatinine urine	115.7 ± 66.6	114.1 ± 63.6	114.3 ± 70.1	NS
Fasting insulin	15.5 ± 17.2	15.7 ± 16.02	16 ± 22.2	NS
Albumin creatinine ratio	80.54 ± 142.9	1.6 ± 0.5	1.72 ± 0.6	NS

*Note*: BMI: body mass index; AHI: apnea–hypopnea index; NS: not significant (*p*-value > 0.01).

**Table 2:** Distribution of PSG phenotypes.

No.	Categories	Features
1	Polysomnography	12
2	Treatment	6
3	Questionnaires	39
	Total	57

**Table 3:** Feature importance analysis: top 10 features.

Rank	Feature Name	Description
1	shq_anxiety	Self-reported physician diagnosis of anxiety disorder
2	mh_norm	General mental health subscale normal score
3	slp_eff	Sleep Efficiency: Proportion of estimated sleep duration in in-bed period from type III home sleep test
4	agg_ment	Summary component score of mental health
5	bmi	Body mass index (BMI)
6	sf36_mcs	SF-36: Mental component summary score (standardized)
7	avgseatedpuls	Average seated radial pulse rate
8	nreadings	Number of clinical data readings, including vital signs or ambulatory blood pressure measurements
9	semsa_oe_nmiss	Number of missing items from the outcome expectancies subscale of the Self-Efficacy Measure of Sleep Apnea
10	cal_total	Total score of the Calgary Sleep Apnea Quality of Life Index (SAQLI)

**Table 4:** Performance evaluation.

Models	Datasets	Precision	Recall	F1-Score	Accuracy
RF	Training set	85.0	85.9	85.5	85.2
	Validation set	83.1	83.4	83.6	83.0
	Test set	80.6	75.2	77.4	80.6
XGBoost	Training set	83.3	83.9	83.5	83.2
	Validation set	80.6	79.4	79.5	81.0
	Test set	82.3	78.4	80.3	80.6
LightGBM	Training set	86.6	83.2	85.1	83.3
	Validation set	80.4	78.0	80.3	77.4
	Test set	81.5	76.9	79.8	74.1
CatBoost	Training set	85.2	85.6	85.3	85.0
	Validation set	71.4	72.5	74.5	82.6
	Test set	83.8	75.7	69.9	74.1
ANN [32]	Training set	75.0	66.8	75.1	81.8
	Validation set	51.6	58.3	55.5	71.7
	Test set	45.4	41.6	43.4	68.1

**Table 5:** Optimized parameters for explainable AI models in depression prediction.

Model	Parameter	Parameter Tested	Optimized Parameters
RF	n_estimators	100, 200, 300, 400, and 500	200
	max_depth	3, 4, 5, 6, and 7	5
XGBoost	n_estimators	100, 200, 300, 400, and 500	300
	learning_rate	0.01, 0.1, 0.2, and 0.3	0.1
	max_depth	3, 4, 5, and 6	5
LightGBM	n_estimators	100, 200, 300, 400, and 500	400
	learning_rate	0.01, 0.05, 0.1, 0.2, and 0.3	0.1
	max_depth	3, 4, 5, and 6	5
Catboost	n_estimators	100, 200, 300, 400, and 500	300
	learning_rate	0.01, 0.05, 0.1, 0.2, and 0.3	0.1
	depth	3, 4, 5, and 6	6

**Table 6:** Comparative evaluation of the proposed method with the existing similar approaches.

Study	Algorithms	Accuracy	Precision	Recall	F1-Score	Year
Amita et al. [17]	XGBoost	97.6	95.5	99.8	97.6	2020
Wang et al. [18]	RF, LightGBM, and CatBoost	69.8	70.0	70.1	70.0	2022
Singh et al. [19]	SVM	94.0	94.0	94.0	94.0	2022
Hassan et al. [14]	SVM-RFE	93.5	94.6	95.9	95.2	2024
Li et al. [15]	RF	87.4	91.7	80.8	—	2024
Fan et al. [16]	RF, LightGBM, XGBoost, and Decision Tree	99.9	99.9	99.9	99.9	2024
This study	RF	85.0	85.0	85.0	85.0	2024
	XGBoost	83.0	83.0	83.0	83.0	
	LightGBM	86.0	83.0	85.0	83.0	
	CatBoost	85.0	85.0	85.0	85.0	

## Data Availability

https://sleepdata.org/datasets/bestair, accessed on 3 May 2023.
